# Supplementary figures and images for: Control of mitochondrial dynamics by the metabolic regulator dPGC1 limits Yorkie-induced oncogenic growth in Drosophila
Source: PLoS Biol. 2025 Dec 4;23(12):e3003523. doi: 10.1371/journal.pbio.3003523 (PMC12697956; doi:10.1371/journal.pbio.3003523)

# S1 Fig. dPGC1 Mutant Wing Discs

*yw*

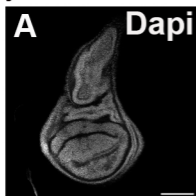

*dPGC1<sup>1</sup>*

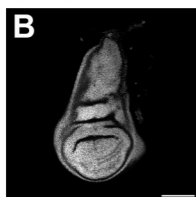

**C**

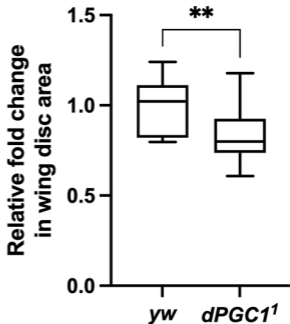

Supplement: S1 Fig — (A, B) Confocal micrographs of Drosophila wing imaginal discs from the following genotypes: yw/yw (control, A) and dPGC11/dPGC11 (mutant, B). DAPI labels the DNA and is shown in grayscale. Scale bars, 100 µm. (C) Quantification of wing disc area of the genotypes in A and B. Wing disc size was normalized to the mean area of the control (yw/yw). Statistical signiﬁcance was determined using an unpaired t test (n = 16 [yw], n = 13 [dPGC11]). **p < 0.01. The data underlying this graph can be found in S2 Data. (PDF) [file pbio.3003523.s001.pdf]

S2 Fig. UAS-Driven Transgene Efficiency

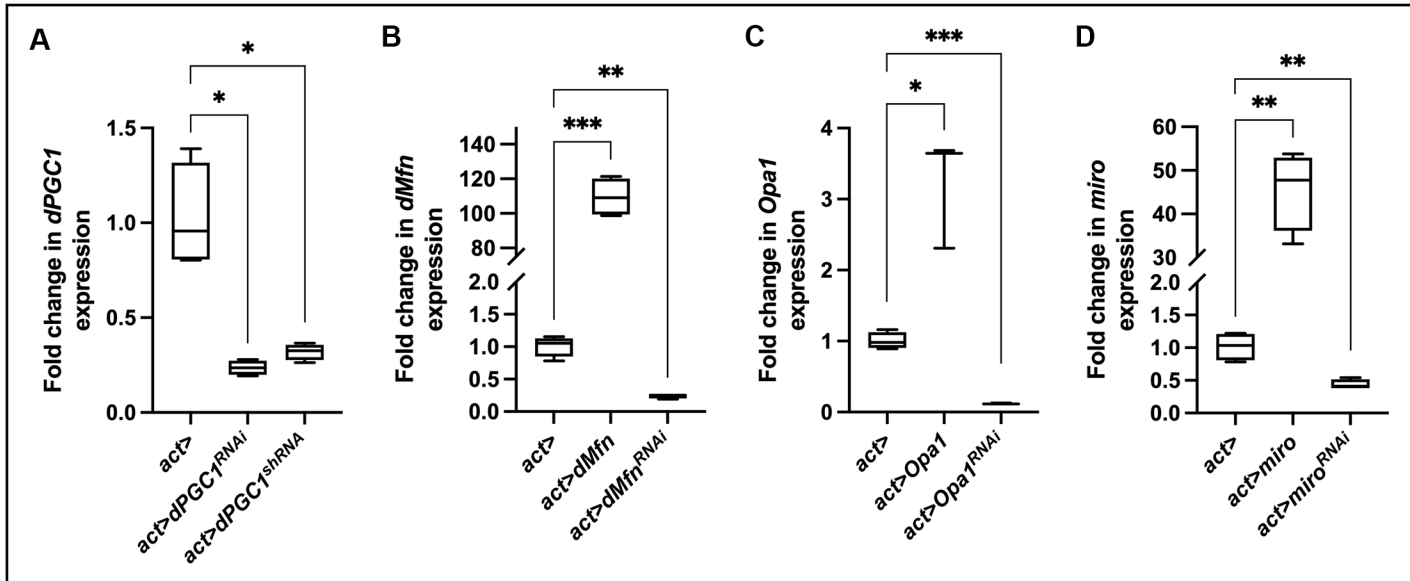

Supplement: S2 Fig — mRNA quantification by qPCR of the transgenes used to modulate the genes dPGC1 (A), dMfn (B), Opa1 (C), and miro (D). The transgenes were expressed under the control of the act-Gal4 driver and whole larvae were used for the analysis. The control genotype is act-Gal4/+. Each genotype was run in three to five biological replicates. RP49 was used as the housekeeping gene. Statistical signiﬁcance was determined using unpaired t tests with or without Welch’s correction depending on whether variances were significantly different or not, respectively. *p < 0.05, **p < 0.01, ***p < 0.001. Additional information about these transgenes is shown in S1 Data. The data underlying all the graphs in the figure can be found in S2 Data. (PDF) [file pbio.3003523.s002.pdf]

S3 Fig. Yki Expression and Activity in Discs with Reduced dPGC1

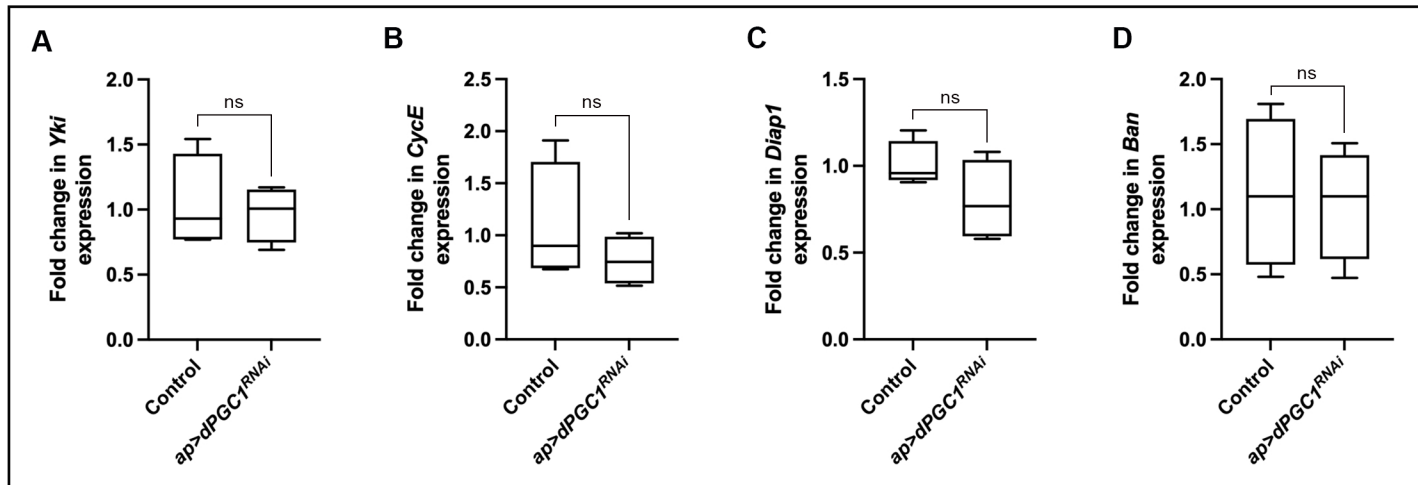

Supplement: S3 Fig — mRNA quantification by qPCR of Yki (A) and the Yki target genes CycE (B), Diap1 (C), and Ban (D) in ap-Gal4, UAS-GFP, UAS-dPGC1-RNAi wing imaginal discs. The control genotype is ap-Gal4, UAS-GFP, UAS-LacZ. Each genotype was run in four biological replicates. RP49 was used as the housekeeping gene. Statistical signiﬁcance was determined using unpaired t tests. ns, non-significant. The data underlying all the graphs in the figure can be found in S2 Data. (PDF) [file pbio.3003523.s003.pdf]

# S5 Fig. Oncogenic Cooperation Between Yki and dPGC1

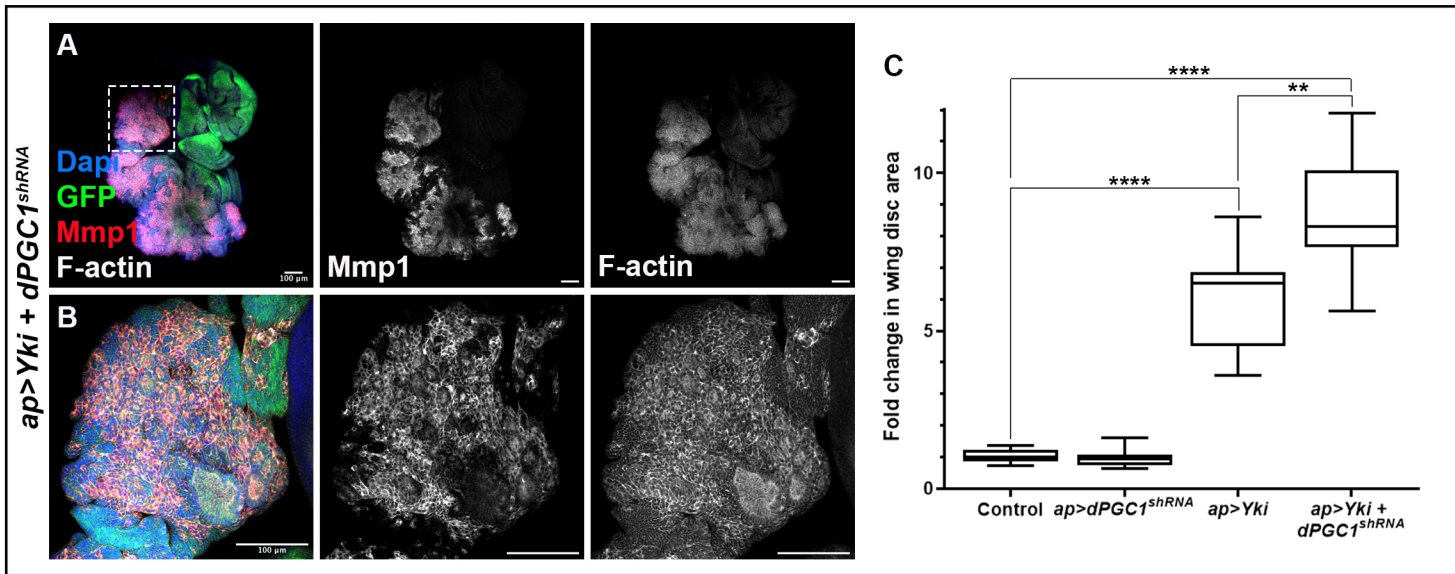

Supplement: S5 Fig — (A, B) Confocal micrograph showing an ap-Gal4, UAS-GFP, UAS-Yki, UAS-dPGC1-shRNA third instar wing imaginal disc. F-actin labels cell polarity and is shown in grayscale. Mmp1 is shown in red. GFP is shown in green. DAPI labels the DNA and is shown in blue. The dashed white box in A indicates the region of the wing disc that is shown as a magnification in B. Scale bars, 100 μm. (C) Quantiﬁcation of GFP-positive area in third instar wing imaginal discs of the indicated genotypes. GFP-positive areas were normalized to the mean of the control (ap > LacZ). Statistical signiﬁcance was determined using unpaired t tests with Welch’s correction (n = 10 [Control], n = 10 [ap > dPGC1-shRNA], n = 10 [ap > Yki], n = 10 [ap > Yki, dPGC1-shRNA]). **p < 0.01, ****p < 0.0001. The data underlying this graph can be found in S2 Data. (PDF) [file pbio.3003523.s005.pdf]

## S6 Fig. Membrane Potential in Yki+dPGC1-RNAi tumors

*ap>Yki*

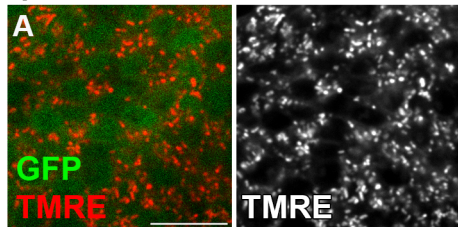

*ap>Yki + dPGC1<sup>RNAi</sup>*

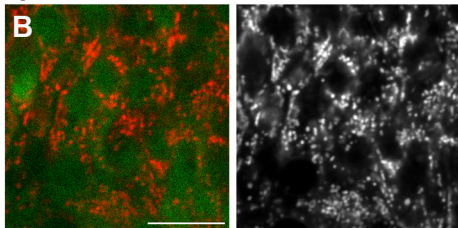

**C**

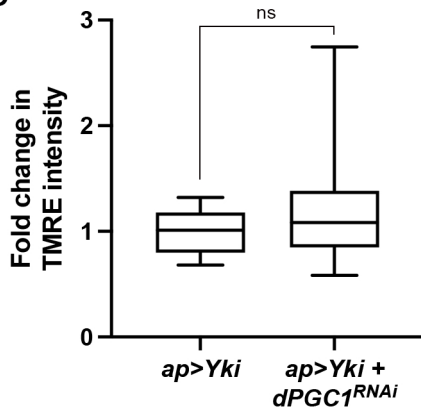

Supplement: S6 Fig — (A, B) Confocal micrographs showing magnifications from tumorous wing discs with the following genotypes: ap-Gal4, UAS-Yki, UAS-GFP, UAS-LacZ (A) and ap-Gal4, UAS-Yki, UAS-GFP, UAS-dPGC1-RNAi (B). GFP is shown in green. TMRE staining is shown in red. Scale bars, 10 μm. (C) Quantification of TMRE intensity in the genotypes in A and B. TMRE intensity was normalized to the mean of the control (ap > Yki). Statistical signiﬁcance was determined using a Mann-Whitney test for non-parametric data (n = 23 [ap > Yki], n = 56 [ap > Yki, dPGC1-RNAi). ns, non-significant. The data underlying this graph can be found in S2 Data. (PDF) [file pbio.3003523.s006.pdf]

S7 Fig. Expression of Genes Controlling Mitochondrial Dynamics in Discs with Reduced dPGC1

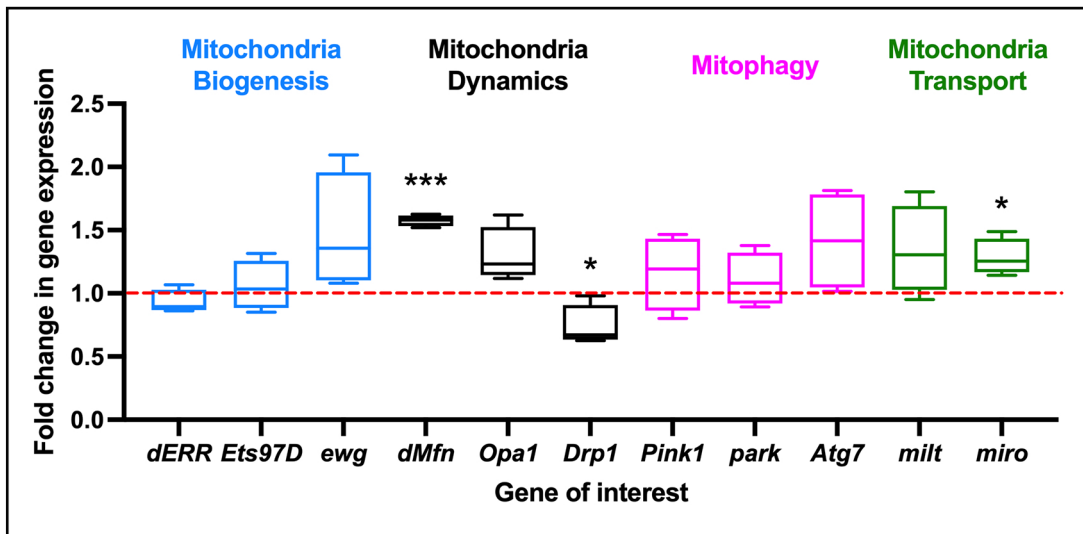

Supplement: S7 Fig — mRNA quantification by qPCR of the indicated genes in ap-Gal4, UAS-GFP, UAS-dPGC1-RNAi wing imaginal discs using ap-Gal4, UAS-GFP, UAS-LacZ as the control genotype. Genes are separated in different categories: mitochondria biogenesis (blue), mitochondria dynamics (black), mitophagy (pink), and mitochondria transport (green). Each genotype was run in four biological replicates. RP49 was used as the housekeeping gene. Statistical signiﬁcance was determined using unpaired t tests with Welch’s correction for parametric data and Mann–Whitney tests for non-parametric data. *p < 0.05, ***p < 0.001. The data underlying this graph can be found in S2 Data. (PDF) [file pbio.3003523.s007.pdf]

S8 Fig. dPGC1 Overexpression in a Context of Yki Upregulation

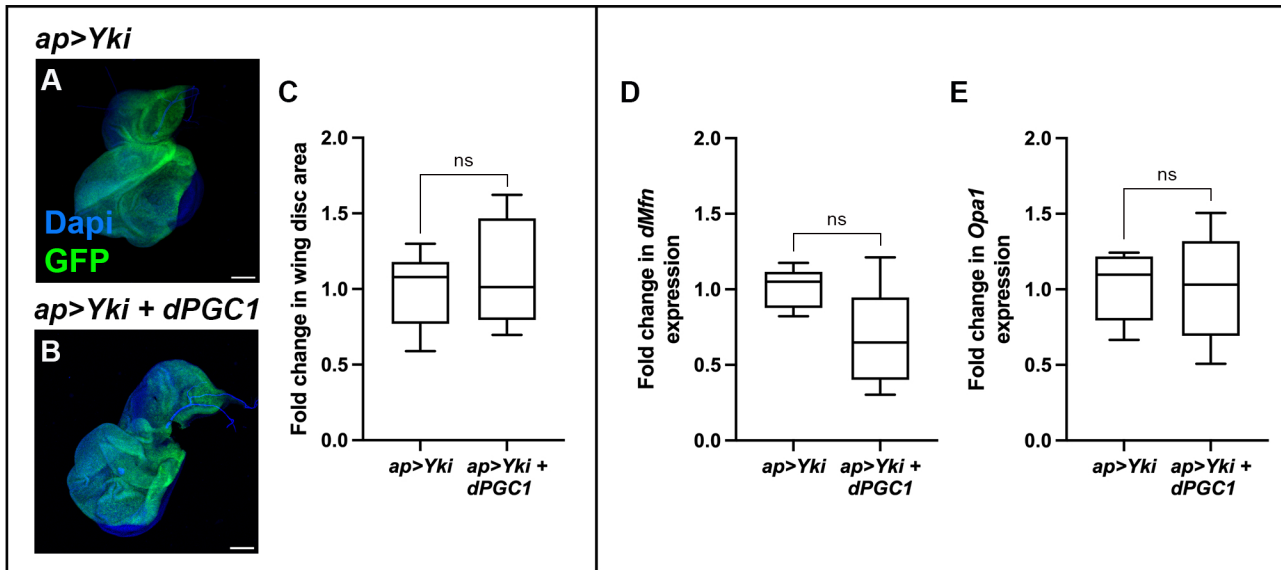

Supplement: S8 Fig — (A, B) Confocal micrographs of wing imaginal discs of the following genotypes: ap-Gal4, UAS-Yki, UAS-GFP, UAS-LacZ (A) and ap-Gal4, UAS-Yki, UAS-GFP, EP-dPGC1 (B). GFP is shown in green. DAPI labels the DNA and is shown in blue. Scale bars, 100 µm. (C) Quantiﬁcation of GFP-positive area in third instar wing imaginal discs of the genotypes in A and B. GFP-positive areas were normalized to the mean of the control (ap > Yki). Statistical signiﬁcance was determined using an unpaired t test (n = 14 [ap > Yki], n = 10 [ap > Yki, dPGC1). ns, non-significant. (D, E) mRNA quantification by qPCR of dMfn (D) and Opa1 (E) in the indicated genotypes. The control genotype is ap > Yki. Each genotype was run in five biological replicates. RP49 was used as the housekeeping gene. Statistical signiﬁcance was determined using unpaired t tests. ns, non-significant. The data underlying all the graphs in the figure can be found in S2 Data. (PDF) [file pbio.3003523.s008.pdf]

S9 Fig. Changes in Mitochondrial Shape upon dMfn, Opa1, and miro Gene Manipulation

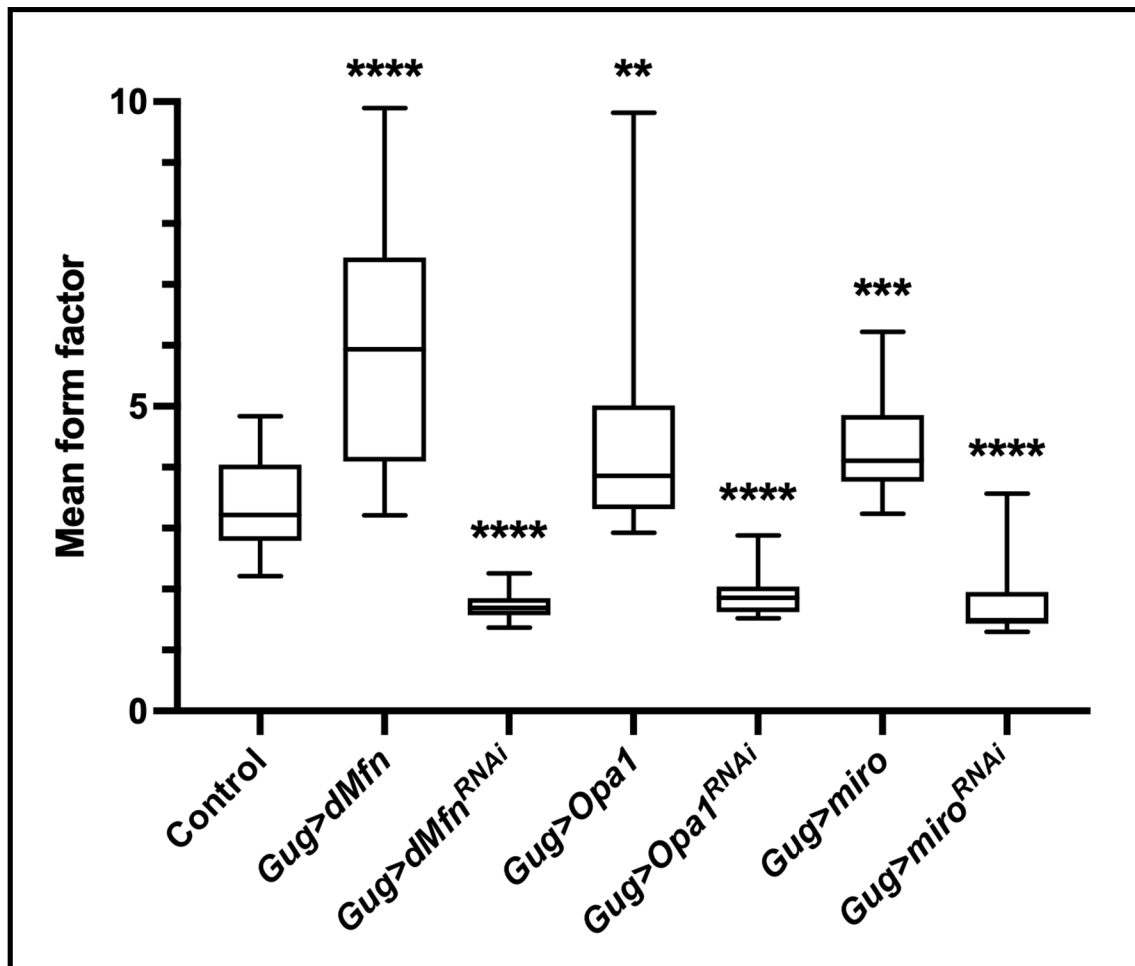

Supplement: S9 Fig — Quantification of mitochondrial mean form factor (shape measure where a value of 1 indicates a round object and values increase with elongation) from confocal micrographs obtained from the peripodial membrane of the following genotypes: Gug-Gal4, UAS-Mito-GFP (Control); Gug-Gal4, UAS-Mito-GFP, UAS-dMfn; Gug-Gal4, UAS-Mito-GFP, UAS-dMfn-RNAi; Gug-Gal4, UAS-Mito-GFP, EP-Opa1; Gug-Gal4, UAS-Mito-GFP, UAS-Opa1-RNAi; Gug-Gal4, UAS-Mito-GFP, UAS-miro; and Gug-Gal4, UAS-Mito-GFP, UAS-miro-RNAi. Statistical signiﬁcance was determined using unpaired t tests with Welch’s correction for parametric data and Mann–Whitney tests for non-parametric data (n = 20 [Control], n = 20 [Gug > dMfn], n = 20 [Gug > dMfn-RNAi], n = 23 [Gug > Opa1], n = 23 [Gug > Opa1-RNAi], n = 20 [Gug>miro], n = 20 [Gug>miro-RNAi]). ** p < 0.01, ***p < 0.001, ****p < 0.0001. The data underlying this graph can be found in S2 Data. (PDF) [file pbio.3003523.s009.pdf]

# S10 Fig. Wing Disc Size upon dMfn and Opa1 Manipulation

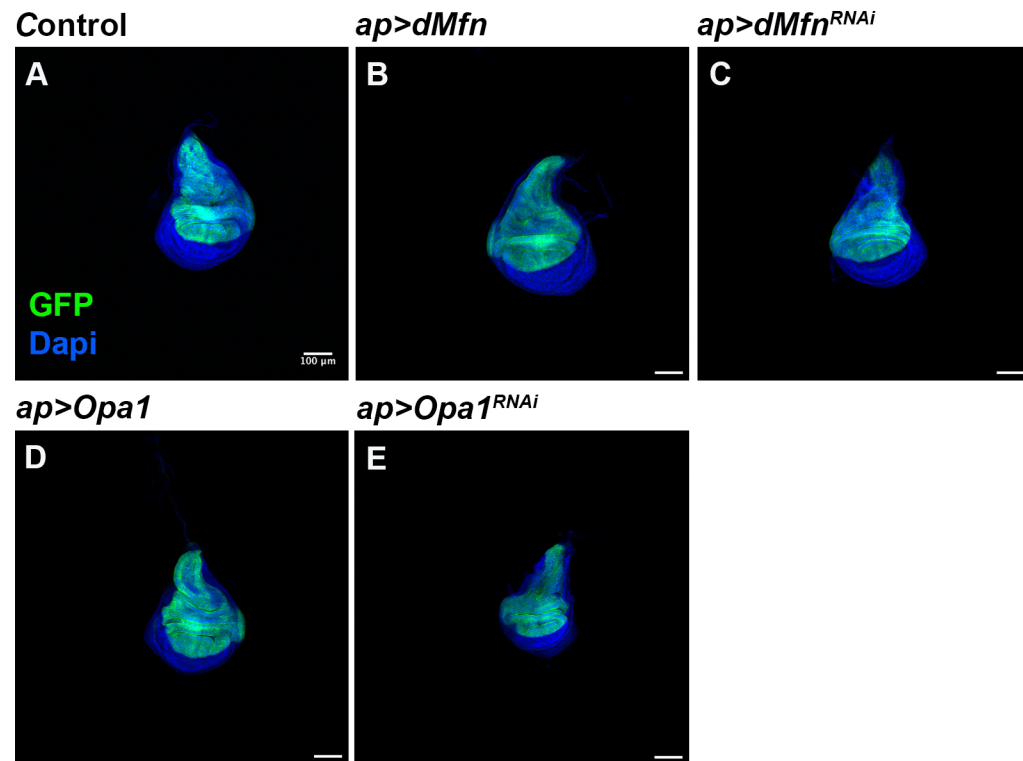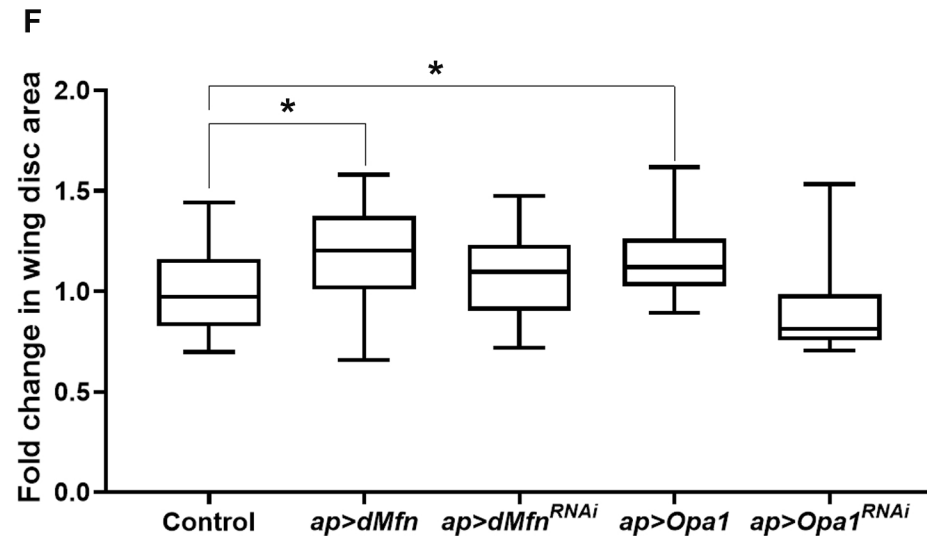

Supplement: S10 Fig — (A–E) Confocal micrographs showing discs of the following genotypes: ap-Gal4, UAS-GFP, UAS-LacZ (A); ap-Gal4, UAS-GFP, UAS-dMfn (B); ap-Gal4, UAS-GFP, UAS-dMfn-RNAi (C); ap-Gal4, UAS-GFP, EP-Opa1 (D); and ap-Gal4, UAS-GFP, UAS-Opa1-RNAi (E). GFP is shown in green. DAPI labels the DNA and is shown in blue. Scale bars, 100 μm. (F) Quantiﬁcation of GFP-positive area in third instar wing imaginal discs of the genotypes shown in A–E. GFP-positive area was normalized to the mean of the control (ap > LacZ). Statistical signiﬁcance was determined using unpaired t tests with Welch’s correction for parametric data and Mann–Whitney tests for non-parametric data (n = 18 [Control], n = 18 [ap > dMfn], n = 20 [ap > dMfn-RNAi], n = 20 [ap > Opa1], n = 19 [ap > Opa1-RNAi]). *p < 0.05. The data underlying this graph can be found in S2 Data. (PDF) [file pbio.3003523.s010.pdf]

# S11 Fig. Miro Does Not Affect Yki-Driven Tumors

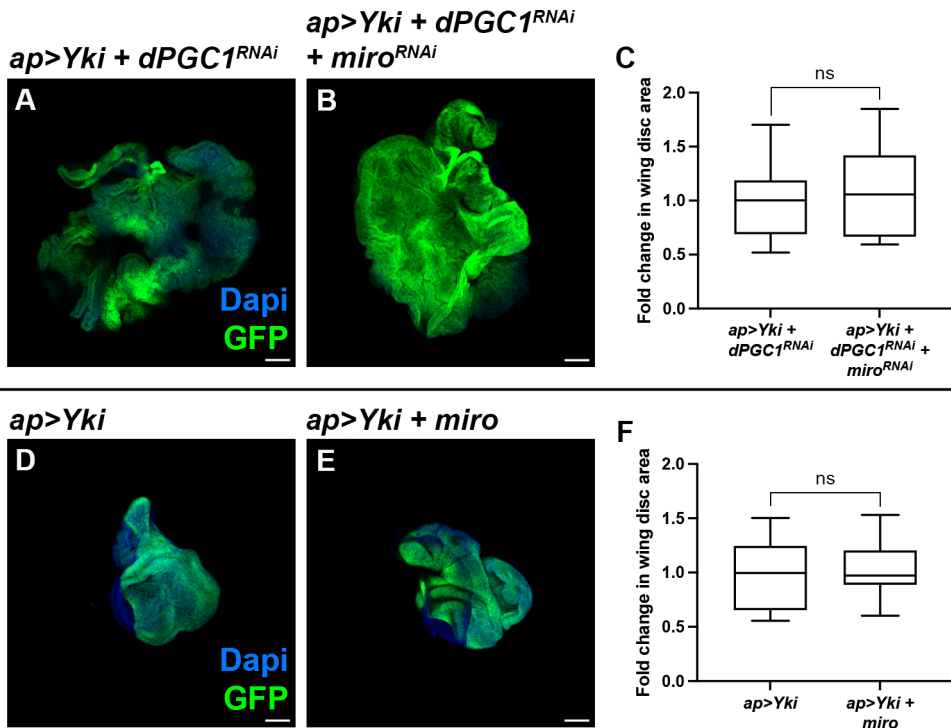

Supplement: S11 Fig — (A, B) Confocal micrographs of wing imaginal discs of the following genotypes: ap-Gal4, UAS-Yki, UAS-GFP, UAS-dPGC1-RNAi (A) and ap-Gal4, UAS-Yki, UAS-GFP, UAS-dPGC1-RNAi, UAS-miro-RNAi (B). GFP is shown in green. DAPI labels the DNA and is shown in blue. Scale bars, 100 µm. (C) Quantiﬁcation of GFP-positive area in wing imaginal discs of the genotypes in A and B. GFP-positive areas were normalized to the mean of the control (ap > Yki, dPGC1-RNAi). Statistical signiﬁcance was determined using a Mann–Whitney test (n = 22 [ap > Yki, dPGC1-RNAi], n = 26 [ap > Yki, dPGC1-RNAi, miro-RNAi]). ns, non-significant. (D, E) Confocal micrographs of wing imaginal discs of the following genotypes: ap-Gal4, UAS-Yki, UAS-GFP, UAS-LacZ (D) and ap-Gal4, UAS-Yki, UAS-GFP, UAS-miro (E). GFP is shown in green. DAPI labels the DNA and is shown in blue. Scale bars, 100 µm. (F) Quantiﬁcation of GFP-positive area in wing imaginal discs of the genotypes in D and E. GFP-positive areas were normalized to the mean of the control (ap > Yki). Statistical signiﬁcance was determined using an unpaired t test (n = 21 [ap > Yki], n = 25 [ap > Yki, miro]). ns, non-significant. The data underlying all the graphs in the figure can be found in S2 Data. (PDF) [file pbio.3003523.s011.pdf]

S13 Fig. UAS-Dap-RNAi Transgene Efficiency

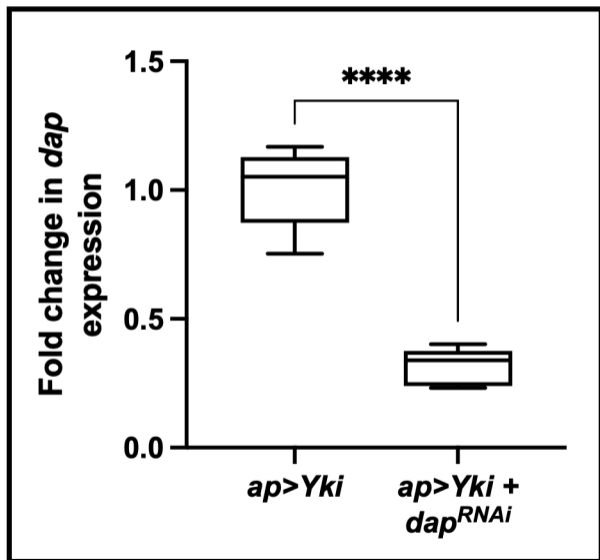

Supplement: S13 Fig — mRNA quantification by qPCR of dap in wing imaginal discs of the following genotypes: ap-Gal4, UAS-Yki, UAS-GFP, UAS-LacZ and ap-Gal4, UAS-Yki, UAS-GFP, UAS-dap-RNAi. The control genotype is ap > Yki. Each genotype was run in five biological replicates. RP49 was used as the housekeeping gene. Statistical signiﬁcance was determined using an unpaired t test. ****p < 0.0001. The data underlying this graph can be found in S2 Data. (PDF) [file pbio.3003523.s013.pdf]

S14 Fig. Cyclin E in Wing Imaginal Discs of dPGC1 Mutant Flies

*yw*

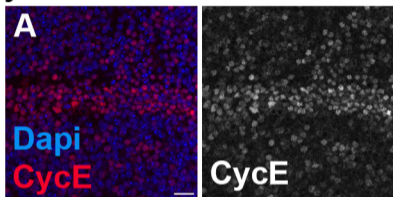

*dPGC1<sup>1</sup>*

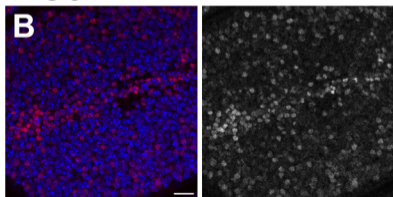

**C**

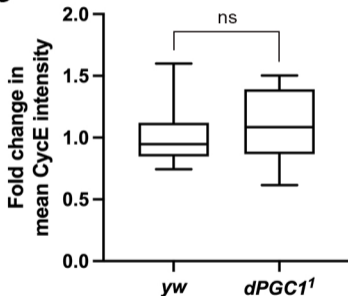

Supplement: S14 Fig — (A, B) Confocal micrographs showing the wing pouch of imaginal discs of the following genotypes: yw/yw (control, A) and dPGC11/dPGC11 (mutant, B). Cyclin E is shown in red. DAPI labels the DNA and is shown in blue. Scale bars, 10 µm. (C) Quantification of Cyclin E protein mean intensity of the genotypes in A and B. Cyclin E intensity was normalized to the mean intensity of the control (yw/yw). Statistical signiﬁcance was determined using an unpaired t test (n = 22 [yw], n = 13 [dPGC11]). ns, non-significant. The data underlying this graph can be found in S2 Data. (PDF) [file pbio.3003523.s014.pdf]

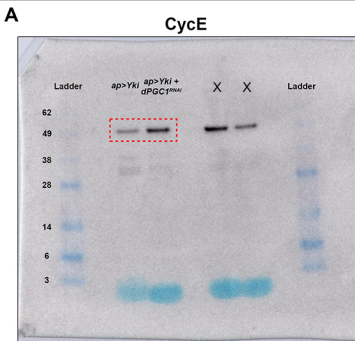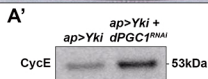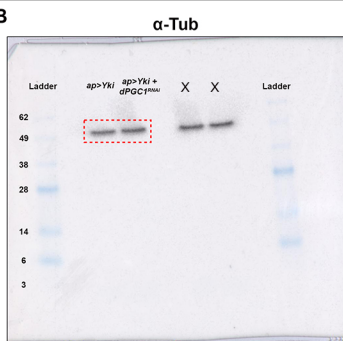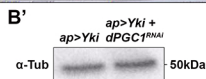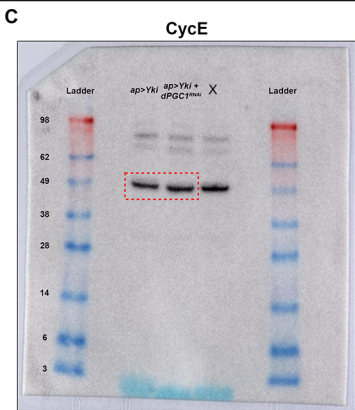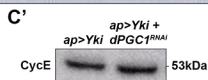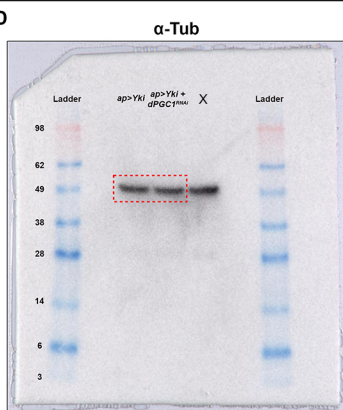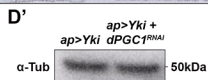

Supplement: S1 Raw Images — These images show the original western blot membranes used to analyze Cyclin E and α-Tubulin protein levels in wing imaginal discs from Drosophila genotypes with Yki overexpression and dPGC1 depletion. Molecular weight markers are indicated, and the relevant lanes are annotated to match the experimental conditions described in the manuscript. Lanes not relevant to this study have been labeled as “X” to clearly distinguish them from the experimental samples. These raw data images are provided in compliance with PLOS Biology guidelines for transparency and reproducibility. (PDF) [file pbio.3003523.s017.pdf]
