# Supplementary material for: Control of mitochondrial dynamics by the metabolic regulator dPGC1 limits Yorkie-induced oncogenic growth in Drosophila
Source: PLoS Biol. 2025 Dec 4;23(12):e3003523. doi: 10.1371/journal.pbio.3003523 (PMC12697956; doi:10.1371/journal.pbio.3003523)

S4 Fig. *dPGC1* Depletion Does Not Cooperate with the oncogenes *EGFR* and *InR*

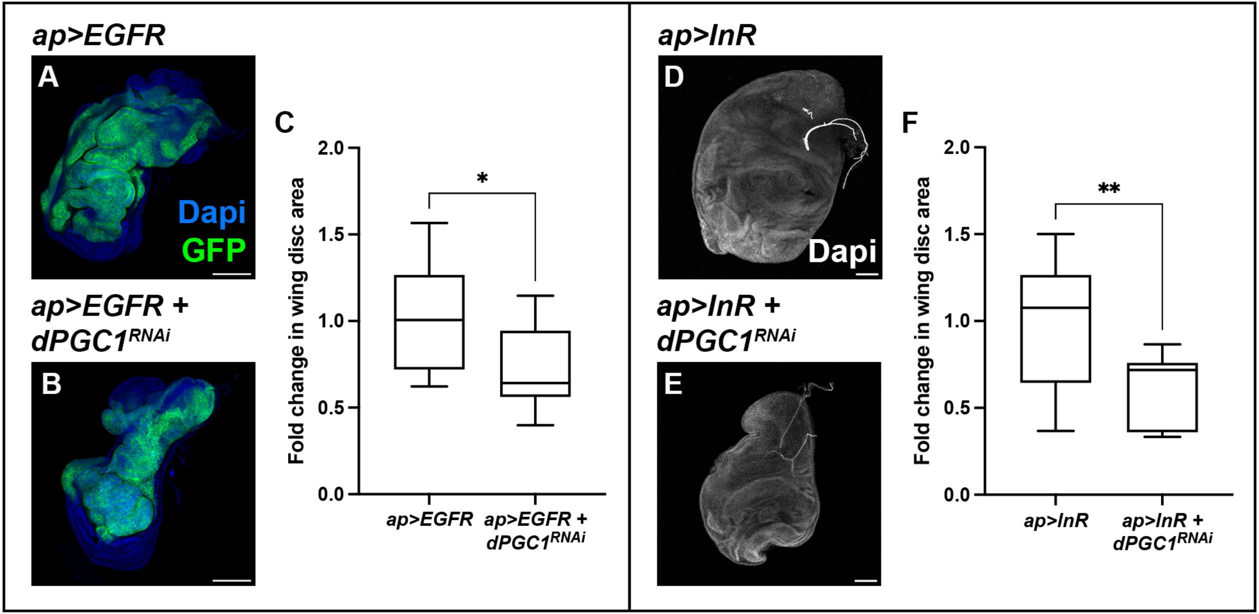

Supplement: S4 Fig — (A, B) Confocal micrographs of wing imaginal discs of the following genotypes: ap-Gal4, UAS-EGFR, UAS-GFP, UAS-LacZ (A) and ap-Gal4, UAS-EGFR, UAS-GFP, UAS-dPGC1-RNAi (B). GFP is shown in green. DAPI labels the DNA and is shown in blue. Scale bars, 100 µm. (C) Quantification of wing disc area (GFP-positive area) of the genotypes in A and B. GFP-positive area was normalized to the mean of the control (ap>EGFR). Statistical significance was determined using an unpaired t test (n = 11 [ap>EGFR], n = 11 [ap>EGFR, dPGC1-RNAi]). *p < 0.05. (D, E) Confocal micrographs of wing imaginal discs of the following genotypes: ap-Gal4, UAS-InR, UAS-GFP (GFP not shown) (D) and ap-Gal4, UAS-InR, UAS-dPGC1-RNAi (E). DAPI labels the DNA and is shown in grayscale. Scale bars, 100 µm. (F) Quantification of wing disc area (DAPI-positive area) of the genotypes in D and E. DAPI-positive area was normalized to the mean of the control (ap>InR). Statistical significance was determined using an unpaired t test (n = 19 [ap>InR], n = 11 [ap>InR, dPGC1-RNAi]). **p < 0.01. The data underlying all the graphs in the figure can be found in S2 Data. (PDF) [file pbio.3003523.s004.pdf]
