# Supplementary material for: Control of mitochondrial dynamics by the metabolic regulator dPGC1 limits Yorkie-induced oncogenic growth in Drosophila
Source: PLoS Biol. 2025 Dec 4;23(12):e3003523. doi: 10.1371/journal.pbio.3003523 (PMC12697956; doi:10.1371/journal.pbio.3003523)

S12 Fig. Representative Whole Western Membrane Images of Cyclin E Protein Levels in Yki + dPGC1-RNAi Tumors

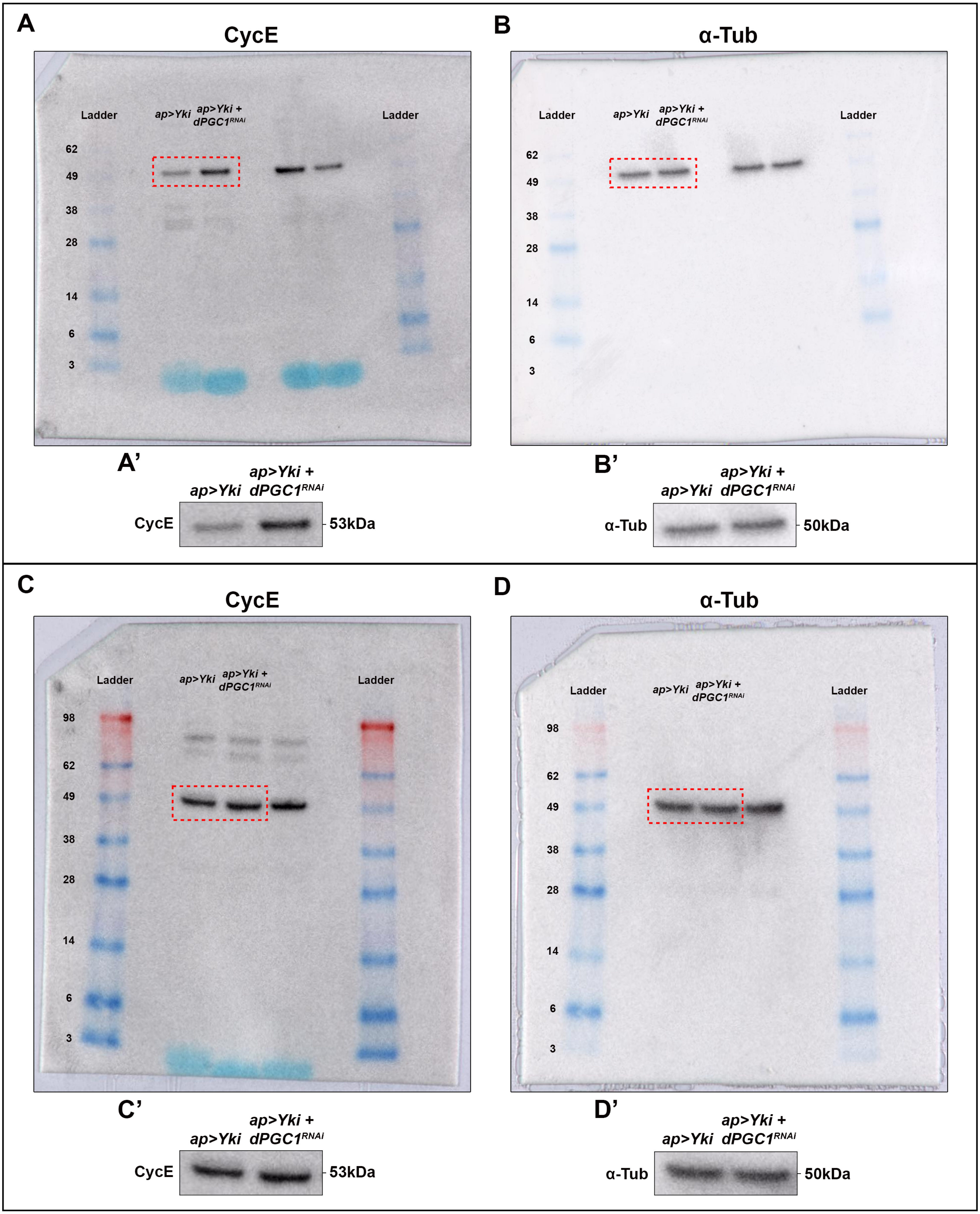

Supplement: S12 Fig — (A–D) Examples of two western blot membranes to analyze protein levels of Cyclin E (A and C) and α-Tubulin (B and D) in wing imaginal discs of the following genotypes: ap-Gal4, UAS-Yki, UAS-GFP, UAS-LacZ; and ap-Gal4, UAS-Yki, UAS-GFP, UAS-dPGC1-RNAi. The molecular weights (in kDa) of the visible bands of the ladder are indicated at the left of each membrane. The dashed red boxes in A–D indicate the regions of the membranes that are shown as magnifications in A′–D′, respectively. Note that the western blot membrane of panels A and B corresponds to the one shown in Fig 6B and therefore panels A′ and B′ are the same as those in Fig 6B. The membranes shown here include additional genotypes not relevant to this study. Only the lanes corresponding to the relevant samples are indicated. (PDF) [file pbio.3003523.s012.pdf]
