# Supplementary material for: Control of mitochondrial dynamics by the metabolic regulator dPGC1 limits Yorkie-induced oncogenic growth in Drosophila
Source: PLoS Biol. 2025 Dec 4;23(12):e3003523. doi: 10.1371/journal.pbio.3003523 (PMC12697956; doi:10.1371/journal.pbio.3003523)
Supplement: Attachment — Submitted filename: 153172_1_rebuttal_3487646_t1jgxz.pdf [file pbio.3003523.s018.pdf]

Full Revision

Manuscript number: RC-2024-02816
Corresponding author(s): Héctor Herranz

1. General Statements

We thank the reviewers for their thoughtful and constructive feedback, which has been instrumental in improving the overall quality of our manuscript.

In response, we have undertaken a substantial revision that includes new experimental data, refined analyses, and clearer presentation of our findings. Specifically, we have addressed concerns about RNAi efficiency and protein-level validation, expanded our genetic models to include loss-of-function contexts, and clarified the interpretation of mitochondrial morphology using both confocal and electron microscopy. We also incorporated new data on Cyclin E regulation and mitochondrial membrane potential to strengthen the mechanistic link between dPGC1 depletion and Yki-driven tumorigenesis. These revisions not only address the specific points raised by the reviewers but also enhance the coherence and impact of the study. We are confident that the revised manuscript presents a more robust and compelling case for the role of dPGC1 as a context-dependent tumor suppressor and that it will be of broad interest to the fields of developmental biology, cancer metabolism, and mitochondrial dynamics.

Point-by-point description of the revisions

Reviewer #1 (Evidence, reproducibility and clarity (Required)):
Sew et al. examine the master regulator of mitochondrial biogenesis, dPGC1, in the context of Drosophila wing and larval development. They primarily use confocal imaging to probe the interplay between dPGC1 and an overactive Hippo pathway, driven by overexpression of the main effector protein, Yki. In their study, they find that tumors, driven by overactivity of Yki grow larger when dPGC1 is downregulated, implicating the mitochondrial biogenesis pathway in tumor suppression. Furthermore, in the context of Yki overexpression, they find that levels of Mfn or Opa1 modulate tumor size. Lastly, they show a role of cyclin E in controlling the size of tumors formed by Yki OE + dPGC1 RNAi. The potential role of dPGC1 as a tumor suppressor is interesting because it highlights an emerging recognition of mitochondria in the aetiology of cancer. However, before publication, much of the data in this manuscript should be strengthened by a refinement in the methods/analysis and an increase in orthogonal approaches.

We addressed concerns regarding RNAi efficiency and wing development by incorporating data from a dPGC1 mutant allele and using a ubiquitous driver for qPCR validation of transgene efficiency. We clarified the rationale for EM use. The manuscript now avoids overinterpretation

of mitochondrial morphology and focuses on fusion-specific regulators. We also revised the narrative arc to maintain coherence and added loss-of-function models to support our conclusions.

Below, we address each of the reviewer's points in detail; our responses are highlighted in blue for clarity.

Major comments:

The authors indicate that for example, in lines127-28, that neither downregulating or overexpressing dPGC1 affects wing size. However, the quantification in Fig. 1C shows a significant decrease in wing size following RNAi treatment. This decrease is modest, but it is nevertheless significant. It is worth pointing out, too, that the efficiency of the RNAi in Fig. S1C suggests that the conclusions drawn are premature. While a roughly 55% drop in mRNA levels may be statistically significant, it is unclear whether this drop in transcripts corresponds to a commensurate depletion of protein. Moreover, it is unclear, in this context, how much dPGC1 may indeed be necessary to drive a relatively normal program of mitochondrial biogenesis in wing development. To obtain a clear result, it is necessary to show significant depletion of the dPGC1 protein. (Ultimately, if it is the case that dPGC1 is unnecessary for wing development and function, a more coherent line of inquiry would be to find out the reason for this rather than to pivot the story to studying tumorigenesis in larva.)

We agree that the interpretation of the RNAi efficiency data requires clarification.

The qPCR analysis shown in former Fig. S1C was performed using wing discs from flies expressing UAS-dPGC1-RNAi under the control of the MS1096-Gal4 driver. However, as shown in current Fig. 1C, MS1096-Gal4 is not expressed uniformly across the wing disc. Some regions remain RFP-negative, indicating that the RNAi construct is not active in all cells. As a result, the measured mRNA levels likely underestimate the true knockdown efficiency. This is because the qPCR includes mRNA from both RNAi-expressing and non-expressing cells, diluting the apparent reduction in transcript levels.

To address this limitation and more accurately assess RNAi efficiency, we repeated the qPCR analysis using a ubiquitous driver (actin-Gal4) to ensure uniform expression of the RNAi construct. Under these conditions, we observed a more substantial knockdown, with dPGC1 mRNA levels reduced to approximately 25% of control levels (this is shown in current Fig S2). This result indicates that the RNAi line is more effective than initially suggested by the MS1096-Gal4-based analysis.

To complement our RNAi-based analysis, we additionally used a mutant strain carrying a characterized allele of dPGC1 ($dPGC1^{1}$, also known as $dPGC1^{KG08646}$; see FlyBase: https://flybase.org/reports/FBal0148128). This genetically distinct approach allowed us to validate and strengthen our findings regarding dPGC1 function. Flies homozygous for this allele exhibited a modest but statistically significant reduction in both wing disc and adult wing size.

These results support the conclusion that dPGC1 is required for normal wing growth and development. The new data are now included in Figure 1 and referenced in the main text (lines 144-153).

Additionally, as suggested by the reviewer, we have revised the relevant section to maintain a coherent line of inquiry. The updated text can be found in lines 163–172.

In Figure 3H-K, it is not clear why the authors used electron microscopy to evaluate mitochondrial morphology. The very good confocal images in Figure 3C-G show a clear change in mitochondrial morphology following the knockdown of Mfn, Opa1, and Miro. While it is clear from the electron micrographs in Figure H that the mitochondria are enlarged, it is not obvious that this increase in length is a result of increased mitochondrial fusion. Indeed, if the mean form factor were used to quantify the shape, it is likely that in both conditions, the value would be close to 1, indicating more of a round object, and it not obvious whether there would be a difference between the Yki OE versus the YkI OE + dPGC1 RNAi. Therefore, from this data alone, it cannot be concluded that the YkI OE + dPGC1 RNAi condition leads to mitochondrial hyperfusion.

Our rationale for including electron microscopy (EM) was to overcome specific limitations in imaging mitochondrial morphology within the main epithelium of the wing disc, where Yki-driven tumors arise. These tumors were generated using ap-Gal4, which drives expression specifically in the main epithelium and is not active in the peripodial membrane. This is an important distinction, as the peripodial membrane—used in Figures 3C–G—has a squamous architecture and larger cytoplasmic volume, making it ideal for high-resolution confocal imaging and for assessing the effects of manipulating dMfn, Opa1, and miro. However, because ap-Gal4 is not expressed in the peripodial membrane, this tissue could not be used to analyze mitochondrial morphology in the actual tumorous context.

To directly evaluate mitochondria in the main epithelium, we employed EM, which provides the resolution necessary to visualize ultrastructural changes that are not easily captured by confocal microscopy in this densely packed tissue. While EM does not directly measure fusion events, it allowed us to detect changes in mitochondrial size and shape that support our broader findings.

We acknowledge that mitochondrial enlargement alone does not definitively demonstrate hyperfusion. However, the EM data were interpreted alongside additional evidence: the upregulation of mitochondrial fusion genes (dMfn and Opa1) in Yki + dPGC1-RNAi tumors, and functional data showing that overexpression of these genes promotes fusion in the peripodial membrane. Together, these findings suggest that dPGC1 depletion enhances mitochondrial fusion in Yki-driven tumors.

To further clarify this point, we also imaged mitochondria in the main epithelium using confocal microscopy. However, the resolution was considerably lower than that achieved with EM, limiting our ability to assess fine mitochondrial structures. We have prepared a representative figure for the reviewer (below), showing representative confocal images of wing discs from three

genotypes: (A) ap-Gal4, UAS-GFP (control), (B) ap-Gal4, UAS-Yki, and (C) ap-Gal4, UAS-Yki, UAS-dPGC1-RNAi. We used anti-ATP-synthase (Abcam, ab14748, dilution 1:200), to label the mitochondria for this Figure. Despite the lower resolution, mitochondria in the Yki + dPGC1-RNAi tumors appear elongated (yellow arrows) compared to those in the other conditions, consistent with the changes observed by EM. We believe this example illustrates the limitations of confocal imaging in this tissue and reinforces the need for EM to accurately assess mitochondrial morphology in the tumorous epithelium.

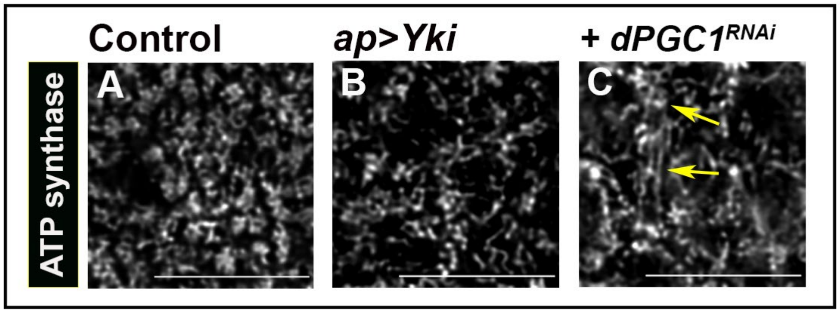

While our EM analyses reveal mitochondrial enlargement in wing discs co-expressing Yki and PGC1-RNAi, we acknowledge that these structural features alone do not conclusively demonstrate mitochondrial hyperfusion. To address this, we have revised the manuscript to avoid overinterpreting the EM data and instead emphasize the functional relevance of mitochondrial fusion regulators such as dMfn and Opa1 in promoting tumor growth.

Taken together, the EM analysis provides structural validation in the tumorous epithelium (Fig 4), while the confocal imaging and functional manipulation of fusion genes in the peripodial membrane offer mechanistic insight (Fig 3). This integrated approach strengthens the conclusion that PGC1 depletion in a Yki-overexpressing context promotes changes in mitochondrial morphology and contributes to tumorigenesis, independent of whether these changes reflect hyperfusion.

Figure 4. refers to changes in mitochondrial fusion and fission in tumor formation; however, the authors do not attempt to alter mitochondrial fission factors, so it is not accurate to mention a role of mitochondrial fission, in this context.

As we did not directly manipulate fission-related factors in our experiments, we agree that it would be inappropriate to draw conclusions about the role of mitochondrial fission in this context. Our revised figure (current Fig 5) and accompanying text now focus exclusively on the effects of mitochondrial fusion and the genes directly involved in regulating this process.

It must be noted, too, that the authors have not demonstrated that their genetic interventions have actually affected mitochondrial morphology in these experiments. As noted in the previous figure, the Yki OE + dPGC1 RNAi condition showed enlarged mitochondria, but not necessarily hyperfused organelles. Therefore, the downregulation of Mfn or Opa1 in this set of experiments may not necessarily have altered mitochondrial morphology. Perhaps suppression of Mfn or

Opa1 would normalize the areas of these evidently swollen mitochondria, but this is unclear without images. Furthermore, it should be appreciated that both Opa1 and Mfn exhibit pleiotropic attributes - e.g., Opa1 not only regulates IMM fusion, but it also modulates the shape and tightness of cristae membranes, specialized sites of oxidative phosphorylation as well as sequestration of cytochrome c, the release of which influences apoptosis (Frezza et al., 2006). At least in mammalian cells, Mfn2 is thought to regulate contacts between mitochondria and endoplasmic reticulum (Naon et al., 2023), which may serve other functions than OMM fusion, such as stabilization of the MAM.

To directly address this point, we performed EM to assess mitochondrial ultrastructure in Yki + dPGC1-RNAi wing disc tumors, with and without dMfn1 downregulation, the most upregulated mitochondrial fusion gene in this tumor context. In Yki + dPGC1-RNAi tumors, mitochondria appeared more elongated, consistent with increased fusion. Upon dMfn1 depletion, we observed a dramatic shift in mitochondrial morphology: mitochondria became larger and more rounded, with disrupted cristae and onion-like structures, indicative of compromised mitochondrial integrity and function (see current Fig. 4).

As the reviewer rightly notes, these morphological changes are consistent with the pleiotropic roles of Mfn and Opa1, which extend beyond outer and inner membrane fusion to include regulation of cristae architecture and ER-mitochondria contacts (Frezza et al., 2006; Naon et al., 2023). We now discuss these broader roles in the revised manuscript (lines 493–497). Taken together, our EM and confocal analyses, combined with targeted genetic manipulations, provide evidence that mitochondrial morphology is indeed altered in response to dPGC1 depletion and fusion gene deregulation in the wing disc.

Figure 5 highlights a connection between dysregulation of mitochondria and Cyclin E, which allows cells to prematurely enter S phase. The data presented here do not offer clarity on whether the enlargement of the tumors results from increase cellular proliferation and/or cell size. The role of the cell cycle adds a layer of complexity to these results, because it is thought that mitochondria undergo fragmentation during the cell cycle to promote an even distribution of the organelle population after mitosis (Taguchi et al., 2007); however, in this manuscript, the authors contend that the downregulation dPGC1 is promoting mitochondrial hyperfusion. It is unclear how and whether cellular division and proliferation would proceed at an accelerated rate in a situation with mitochondrial hyperfusion.

To address this point, we started by analyzing whether Yki + dPGC1-RNAi tumors exhibit increased proliferation compared to tumors expressing Yki alone. We quantified mitotic activity using the phospho-Histone H3 (PH3) marker of mitotic cells and observed a significant increase in PH3-positive cells in the Yki + dPGC1-RNAi condition. These results indicate an elevated proliferation rate in these tumors and are now presented in Fig 2O–Q. In the text, can be found in lines 221-228.

We agree with the reviewer that our findings challenge the conventional view that mitochondrial fragmentation is a prerequisite for mitosis, as we observe increased expression of gene

promoting mitochondrial fusion in the context of dPGC1 downregulation alongside signs of accelerated cell cycle entry. It is important to note that we also show that the levels of the oncogene Cyclin E, a key driver of cell cycle progression and S-phase entry, were elevated in Yki + dPGC1-RNAi tumors compared to those expressing Yki alone, suggesting that the increased proliferation observed is at least in part driven by enhanced cycle activity. To further probe Cyclin E's role, we used the CycE-05306 heterozygous mutant allele, which reduces Cyclin E levels by ~50% without affecting normal development. Notably, this partial reduction strongly suppressed tumor growth in the Yki + dPGC1-RNAi background (Fig 6), underscoring Cyclin E's functional importance in supporting oncogenic growth in this context.

These findings support the notion that defects in the expression of mitochondrial genes involved in mitochondrial morphology induced by dPGC1 depletion do not impair but rather coincide with accelerated cell division.

Minor comments:

Lines 69-72 contrast the roles of PGC1α and β. It is not clear whether the comparison is of their respective roles in cancer or in normal physiology. In either case, it is important to note that PGC1β has been shown to drive mitochondrial fusion as well as biogenesis through its control of MFN2, among other factors (Liesa et al., 2008).

In response, we have clarified the comparison between PGC1α and PGC1β in the introduction to specify that it refers to their roles in cancer. Additionally, we now acknowledge that PGC1β has been shown to promote mitochondrial biogenesis and fusion, notably through the regulation of MFN2, as demonstrated by Liesa et al. (2008). This reference has been added to provide a more balanced and accurate representation of PGC1β's functions. In the text it can be found in lines 77-81.

Although this study focuses on PGC1, the authors do not seem to site the original literature from the Spiegelman lab.

In response to the reviewer's comment, we have added a new section in the introduction that cites key foundational studies from the Spiegelman lab. This addition can be found in the introduction in lines 68-73.

There are 10-20 grammatical errors throughout the text.

We apologize for this. We have carefully revised the text, and we are very confident those errors have been corrected.

<u>**Referee Cross-commenting**</u>

There is agreement among the referees that the potential role of PGC1 as a tumor suppressor is interesting and significant. However, various aspects of this work require attention prior to

publication. For example, there needs to be a complete knock down of PGC1 to come to any conclusion as to its role in wing development. The methods for analyzing mitochondrial morphology need to be clarified and be consistent with standards in the field of mitochondrial dynamics. Also, the authors need to quantify their Western blots to obtain accurate assessments of protein levels. Generally, the study relies too heavily on overexpression experiments; understanding the potential role of mitochondria in regulating the Hippo pathway should include various knockdown and/or knockout models.

Reviewer #1 (Significance (Required)):

Overall, the authors show an interesting dampening effect of dPGC1 on growth of Yki-driven tumors. This data could be relevant for elucidating how dysregulation of the Hippo signalling pathway can underlie tumorigenesis.

The narrative arc of the study, however, appears to lack a focused line of inquiry. Figure 1 highlights an attempt to modulate Drosophila wing size and/or structure by downregulating dPGC1, but to no effect. Although examination of the efficiency of the RNAi revealed that the transcripts were still present in significant quantities; so, the conclusion that dPGC1 is dispensable for wing formation is premature. To have clarity on this point, it would be necessary to completely knockdown the gene, preferably by showing a total loss of protein. This should be feasible for the authors, since they showed Western blotting in Figure 5A. In any event, it seems that this negative data led the authors to study the Hippo pathway in the larval stage. This transition from Figure 1 to 2 seemed somewhat arbitrary and leads to a rather disjointed sense of the main line of inquiry around dPGC1.

It is important to note, too, that the authors highlight a role of mitochondrial dynamics in the pathway of Yki-driven tumor formation; however, they only directly evaluate mitochondrial dynamics in this context in a single assay, namely, Figure 3H-K, and this quantification is likely inaccurate because the mitochondria in the Yki OE + dPGC1 RNAi condition seem to be substantially enlarged, circular structures. It is critical to keep in mind that mitochondrial enlargement does not necessarily stem from hyperfusion. It could come from a decrease in the activity of Drp1 or result from an imbalance between mitochondrial biogenesis and mitophagy.

As noted in our responses above, we have addressed these concerns by clarifying the limitations of our mitochondrial morphology analysis. Additionally, we have expanded the discussion (lines 498-504) to explicitly acknowledge that mitochondrial enlargement does not necessarily indicate hyperfusion. In that paragraph, we consider alternative explanations such as reduced fission or imbalances in mitochondrial biogenesis and mitophagy, and we outline the need for future studies using dynamic assays and additional markers to more precisely dissect mitochondrial remodeling in Yki-driven tumors.

A marked limitation of this study is the overuse of rather artificial manipulations of transcriptional regulatory pathways. The study would benefit a lot from investigation of the loss of function of components of the Hippo pathway rather than just OE of Yki.

Review COMMONS

We performed additional experiments using Warts (Wts) mutant clones to assess the role of dPGC1 in a loss-of-function context within the Hippo pathway. While our initial analyses were based on Yki overexpression, which allowed us to robustly probe the interaction between Yki and dPGC1, we agree that this approach may not fully reflect physiological conditions. By generating Wts mutant clones, which endogenously activate Yki through loss of upstream inhibition, we were able to evaluate the impact of dPGC1 depletion in a more physiologically relevant setting. These new results confirm and extend our previous findings, showing that dPGC1 limits tissue overgrowth even when Yki is activated through loss of Wts, thereby strengthening the biological relevance of our conclusions.

These results are presented in Fig 2F-I. In the text, those results are presented in lines 181-189.

My expertise is in mitochondrial biology, with specialization in super-resolution imaging, mitochondrial dynamics and membrane architecture. I have also worked in the interface between mitochondrial physiology and cancer. With this perspective, I think that the authors uncover a potentially interesting role of PGC1 as a tumor suppressor.

Full Revision

Reviewer #2 (Evidence, reproducibility and clarity (Required)):

Summary
In this manuscript the authors the investigate the role of the mitochondrial regulatory transcription factor dPGC1 in tissue growth and oncogenic transformation. They show that dPGC1 limits hyperplasia mediated by overexpression of Yki in the Drosophila wing disc, while having no effect on normal growth. dPGC1 depletion in discs overexpressing Yki results neoplastic overgrowth and hyperfused mitochondria, which was dependent on the increased expression of genes involved in promoting mitochondrial fusion. Additionally, the authors show that dPGC1 limits CycE levels post-transcriptionally in Yki tumors.

In the revised version of our manuscript, we have clarified the relationship between our findings and prior work by Nagaraj et al., including new experiments that demonstrate the specificity of dPGC1's role in Yki-driven growth. Specifically, we show that dPGC1 depletion does not enhance tissue overgrowth in EGFR or InR contexts, nor does it affect Yki expression or activity. Furthermore, we tested dPGC1 overexpression in Yki-overexpressing tissues and observed no significant changes in growth or mitochondrial fusion gene expression. Additional controls confirmed that Cyclin E upregulation is specific to the Yki + dPGC1 depletion condition, reinforcing the context-dependent nature of our findings.

Each of the reviewer's comments is addressed below, with our responses highlighted in blue.

Major comments
1) The authors mention several times in passing in the results a manuscript from the Banerjee lab (Nagaraj et al 2012), which shows that many of the genes the authors of the present manuscript show are upregulated upon Yki overexpression + dPGC1-RNAi compared with Yki overexpression alone are in fact upregulated upon Yki overexpression alone compared with control (dMfn/marf, opa1, miro - while interestingly dPGC1 itself is not affected). Nagaraj et al further show that Yki-overexpressing discs have longer mitochondria suggesting increased fusion even in the absence of dPGC1 depletion. The findings from Nagaraj et al should be mentioned explicitly in the introduction and the relationship between this manuscript and the present work clearly outlined in the discussion.

In the revised manuscript, we have now explicitly referenced the findings of Nagaraj et al. (2012) in the Introduction (lines 106-118), Results (lines 355-360) and Discussion (lines 466-468) sections.

In the revised Introduction, we summarize their key observations that Yki overexpression alone upregulates mitochondrial fusion genes such as dMfn and Opa1, and leads to mitochondrial elongation, while not affecting dPGC1 expression.

In the revised Results section, we mention that, building on that work, our study demonstrates that dPGC1 depletion further amplifies this effect, leading to enhanced mitochondrial elongation and tumor growth.

In the revised Discussion, we now explicitly reference the findings by Nagaraj et al. (2012), which demonstrated that Yki overexpression promotes mitochondrial fusion and upregulates key fusion genes. We build upon this work by showing that dPGC1 depletion in a Yki-overexpressing background further enhances mitochondrial fusion gene expression and tumor growth. This supports a model in which dPGC1 acts as a safeguard against Yki-induced mitochondrial remodeling and oncogenesis, reinforcing its role as a context-dependent tumor suppressor.

Importantly, we show that this effect is context-dependent and not observed in otherwise normal tissues, highlighting a sensitized mitochondrial response to Yki activation when dPGC1 is lost. These additions help delineate the novel contribution of our study in identifying dPGC1 as a critical modulator of mitochondrial dynamics and tumorigenesis downstream of Yki.

2) Given that Yki overexpression alone induces mitochondrial fusion and that dMfn/marf and opa1 depletion suppresses Yki-induced overgrowth (Nagaraj et al), does dPGC1 overexpression also suppress Yki-induced overgrowth?

If so, is this correlated with reduction in dMfn/marf and opa1 compared with Yki overexpression alone?

In response, we performed additional experiments to assess whether dPGC1 overexpression influences Yki-driven overgrowth. We also analyzed the expression of mitochondrial fusion genes (dMfn and Opa1) in this context. As shown in new Fig. S8, dPGC1 overexpression in Yki-overexpressing wing discs did not significantly affect tissue growth, nor did it alter the mRNA levels of key fusion regulators, dMfn and Opa1. These findings suggest that the transcriptional upregulation of mitochondrial fusion genes observed upon dPGC1 depletion is not a general consequence of altered dPGC1 levels, but rather a specific response that emerges in the context of Yki activation. We now present and discuss these results in the revised manuscript (lines 278-285), highlighting the sensitized nature of mitochondrial remodeling in an oncogenic environment driven by Yki signaling.

3) One important question raised by this study is: how specific is the effect of dPGC1 depletion to Yki-driven overgrowth? As Yki-driven overgrowth already have increased mitochondrial length, it is possible that Yki-expressing cells are already sensitised to the effects of dPGC1 depletion. Interestingly, Nagaraj et al show that mitochondrial morphology is not affected upon EGFR activation (hyperplasia) or upon scrib and avl depletion (neoplasia). The authors should therefore test if dPGC1 depletion can potentiate the growth of other hyperplasia drivers such as activated EGFR and InR in the wing disc.

We tested whether the growth-suppressive effect of dPGC1 depletion was specific to Yki-driven overgrowth or could also potentiate tissue growth in other oncogenic contexts. Specifically, we downregulated dPGC1 in wing discs overexpressing either EGFR or InR. In both cases, we did not observe any enhancement of tissue overgrowth upon dPGC1 depletion, in contrast to what we observed in Yki-overexpressing discs. These results suggest that the sensitivity to dPGC1

depletion is specific to Yki-driven overgrowth and is not a general feature of hyperplastic growth induced by other oncogenes.

These results are shown in Fig S4 and in lines 195-202.

4) There are a few simple control experiments the authors should provide to clarify the relationship between Yki and dPGC1:
- Are Yki levels affected by dPGC1 depletion?

To address the potential regulation of Yki by dPGC1, we performed quantitative PCR (qPCR) analysis to measure the expression levels of yki and its well-established transcriptional targets—Cyclin E, Diap1, and bantam—in wing discs depleted of dPGC1. As shown in Fig. S3, we did not detect significant changes in the transcript levels of yki or its target genes, suggesting that the enhanced phenotype observed upon dPGC1 depletion is unlikely to be driven by increased Yki expression or activity. These results indicate that dPGC1 does not strongly influence Yki expression or activity. These new results are presented in lines 190-194.

- Does dPGC1 knockdown alone modify the expression of the genes tested in Fig.3A? In other words, is this upregulation specific of the Yki-overexpression context?

We have conducted this analysis, and the results are now presented in new Fig S7. While the trend is similar to that observed in tumors with both Yki depletion and dPGC1 depletion, the magnitude of change is smaller compared to the context of Yki overexpression. This is described in the text in lines 273-277.

- Does dPCG1 knockdown also stabilise CycE in the absence of Yki overexpression or does the stabilisation of CycE occur only in Yki tumors?

To address this, we examined Cyclin E levels in wing imaginal discs mutant for dPGC1 alone. Our analysis did not reveal any detectable changes in Cyclin E levels under these conditions. These findings suggest that the upregulation of Cyclin E is not a general consequence of dPGC1 loss, but rather a feature specific to the context of Yki overactivation. The corresponding data are now included in Fig S14 of the revised manuscript. In the text, it can be found in lines 442-448.

5) Figure 3C-G: it is not clear how the authors can quantify the length of 3D structures like mitochondria from 2D TEM images (unless they have done volume reconstruction from consecutive sections) and no details are provided in the methods. The quantification of mitochondrial length has to be performed rigorously as it is a key part of the paper.

We agree that TEM provides only 2D profiles of 3D mitochondrial structures, and that this does not allow for precise volumetric reconstruction. In our study, we measured the longest axis of mitochondria visible in thin TEM sections, which is a commonly used 2D proxy for mitochondrial length in the literature (e.g., PMID: 36367943 and PMID: 38637532). To avoid

misunderstandings, we have clarified in the Material and Methods section that the reported values represent apparent mitochondrial length in 2D sections, not true 3D length. To enhance the accuracy of these estimates, we measured more than three tissues per genotype, multiple regions per tissue, several cells per region, and various fields of view per cell.

Minor Comments:

1) Line 51: "Mitochondria are highly dynamics organelles." should be "Mitochondria are highly dynamic organelles."

We have corrected that mistake. Thanks!

2) Introduction: the authors should summarise the known physiological functions of PGC1α in order to put their findings in context.

We have added a section in the introduction (lines 66-81) summarizing the known physiological functions of PGC1α

3) lines: 121-3: "...depletion of dPGC1...did not have a major impact on adult wing size and shape (Fig 1B, C)." There is a small but statistically significant difference so the authors should state this in the text.

We have revised the text to acknowledge that dPGC1 depletion leads to a modest but statistically significant reduction in wing size. In addition to the original analysis, we have now included further experiments to strengthen this point. Specifically, we analyzed wings from flies homozygous for the $dPGC1^{1}$ allele (also known as $dPGC1^{KG08646}$; see FlyBase: https://flybase.org/reports/FBal0148128) and confirmed a small but significant reduction in both wing disc and adult wing size compared to controls (this can be found in Fig. 1 and Fig. S1). These results support the conclusion that, although dPGC1 is dispensable for viability and gross morphology, it contributes to normal wing growth. These new results can be found in lines 144-153.

4) Figure 5A (Cyclin E western blot): the authors should show molecular weight markers. In the revised version of our manuscript, we are including the molecular markers as indicated by the reviewer. These can be found in Fig S12.

Reviewer #2 (Significance (Required)):

The manuscript by Sew et al builds on the previous work by Nagaraj et al to explore the role of mitochondrial function in tumors driven by disruption of the Hippo pathway. In particular, the authors identify dPGC1 as a transcription factor that limits Yki-driven mitochondrial fusion and tissue growth. Interestingly, they further show that Yki/PGC1-depleted tumors are highly sensitive to Cyclin E levels, due to post-transcriptional Cyclin E increase. These results further our knowledge of how Yki drives growth and how mitochondria participate in oncogenic

transformation. With appropriate revision as outlined above (for example exploring whether the mechanism proposed is Yki-specific), the manuscript will be of broad interest to developmental and cancer biologists.

Reviewer #3 (Evidence, reproducibility and clarity (Required)):

The manuscript presents compelling evidence that dPGC1 acts as a context-dependent tumor suppressor in Drosophila by modulating mitochondrial dynamics and limiting Yorkie (Yki)-induced oncogenic growth. By leveraging the Drosophila wing imaginal disc as a model, the authors investigate how dPGC1 depletion exacerbates Yki-driven tissue overgrowth, mitochondrial hyperfusion, Cyclin E upregulation, and DNA damage, leading to tumorigenesis. The study provides valuable insights into the interplay between mitochondrial dynamics and cancer, with implications for understanding metabolic regulation in oncogenesis.
While the findings are significant and well-aligned with the field, certain aspects of the experimental design, data presentation, and mechanistic insights require further attention to enhance clarity, reproducibility, and impact. Below, I outline my major concerns and recommendations.

We addressed concerns about RNAi efficiency and protein-level validation with new qPCR data and mutant analysis. We provided EM and confocal evidence of mitochondrial changes. We clarified non-autonomous effects and quantified Mmp1 and F-actin and added data on miro and Opa1 manipulations. Cyclin E quantification was expanded using multiple Western replicates and a validated mutant allele, and we included new data on mitochondrial membrane potential to assess functional consequences.

Our detailed responses to each point raised by the reviewer are provided below and highlighted in blue.

Major Points

1. One point is the knock-down efficiency of dPGC1 on the mRNA level, which is between 30 to >50% (Fig. S1C). This is not too strong, so the question arises how severly the protein levels are affected. If possible, an antibody staining with quantification should be performed. From these data it cannot be concluded dPGC1 is not required for normal development, half the dose could be sufficient. How do wings look like when the ap-GAL4 driver is used for dPGC1 knock-down, as this is the driver used in the subsequent experiments?

Reviewer 1 also raised concerns about the potential inefficiency of the RNAi treatment in revealing a function during normal wing growth. We agree with both reviewers that the interpretation of the RNAi efficiency data requires clarification.

The qPCR analysis shown in former Fig. S1C was performed using wing discs from flies expressing UAS-dPGC1-RNAi under the control of the MS1096-Gal4 driver. However, as shown in current Fig. 1C, MS1096-Gal4 is not expressed uniformly across the wing disc. Some regions remain RFP-negative, indicating that the RNAi construct is not active in all cells. As a result, the measured mRNA levels likely underestimate the true knockdown efficiency. This is because the

qPCR includes mRNA from both RNAi-expressing and non-expressing cells, diluting the apparent reduction in transcript levels.

To address this limitation and more accurately assess RNAi efficiency, we repeated the qPCR analysis using a ubiquitous driver (actin-Gal4) to ensure uniform expression of the RNAi construct. Under these conditions, we observed a more substantial knockdown, with dPGC1 mRNA levels reduced to approximately 25% of control levels (this is shown in current Fig S2). This result indicates that the RNAi line is more effective than initially suggested by the MS1096-Gal4-based analysis.

To complement our RNAi-based analysis, we additionally used a mutant strain carrying a characterized allele of dPGC1 (dPGC1^{1}, also known as dPGC1^{KG08646}; see FlyBase: https://flybase.org/reports/FBal0148128). This genetically distinct approach allowed us to validate and strengthen our findings regarding dPGC1 function. Flies homozygous for this allele exhibited a modest but statistically significant reduction in both wing disc and adult wing size. These results support the conclusion that dPGC1 is required for normal wing growth and development. The new data are now included in Figure 1 and referenced in the main text (lines 144-151).

Unfortunately, we cannot perform antibody staining due to the unavailability of antibodies against dPGC1.

2. How does the wing disc look like when dPGC1 is overepressed together with Yki?

In response, we performed additional experiments to assess whether dPGC1 overexpression influences Yki-driven overgrowth. We also analyzed the expression of mitochondrial fusion genes (dMfn and Opa1) in this context. As shown in new Fig. S8, dPGC1 overexpression in Yki-overexpressing wing discs did not significantly affect tissue growth, nor did it alter the mRNA levels of key fusion regulators, dMfn and Opa1. These findings suggest that the transcriptional upregulation of mitochondrial fusion genes observed upon dPGC1 depletion is not a general consequence of altered dPGC1 levels, but rather a specific response that emerges in the context of Yki activation. We now present and discuss these results in the revised manuscript (lines 278-285), highlighting the sensitized nature of mitochondrial remodeling in an oncogenic environment driven by Yki signaling.

3. In Fig 2D (but also in Fig. 2C) not only cells in the dorsal but also in the ventral comparmtent seem to overproliferate. Either this is a mis-conception or it is a non-autonomous effect from interfering with Yki and dPGC1 in the vertrnal compartment. In either cases, this has to be clarified.

Ventral cells are not labelled by GFP. Fig 3D shows a tumor in which GFP-negative cells are not present, suggesting that they are not overproliferating but rather being eliminated. This phenomenon is consistent with cell competition, a well-characterized process in which transformed or tumorigenic cells outcompete and eliminate neighboring wild-type cells. We have

previously described this behavior in wing disc tumors (PMID: 26853367; DOI: 10.1016/j.cub.2015.12.042), and it likely contributes to the expansion of the tumor mass by removing surrounding normal tissue also in this context.

4. In Fig. 2F-H quantification of Mmp1 and F-actin is missing. Mmp1 is a JNK target, so the authors could do in addition an anti-phospho JNK antibody staining.

In response, we have performed those quantifications. They are now included in Fig 2M, N.

5. In Fig. 3: how does the mitochondrial network look like in the wing disc periopodial epithelium using the Gug>Yki+dPGC1 genotype? Is it similar to Gug>dMfn or Gug>miro?

We attempted to perform this analysis; however, Yki overexpression under the control of Gug-GAL4 resulted in larval lethality, likely due to GAL4 activity in essential tissues such as the central nervous system. As a result, we were only able to induce transgene expression for 24 hours before lethality occurred.

At this early point, no detectable changes in mitochondrial morphology were observed in the peripodial membrane, likely because the duration of transgene expression was insufficient to elicit phenotypic alterations in this specific tissue. Therefore, while we aimed to compare this genotype to Gug>dMfn and Gug>miro, the technical limitations prevented a conclusive analysis.

We have prepared a representative figure for the reviewer (below), showing representative confocal images of wing discs showing mito-GFP and Dapi in the three genotypes indicated in the Fig.

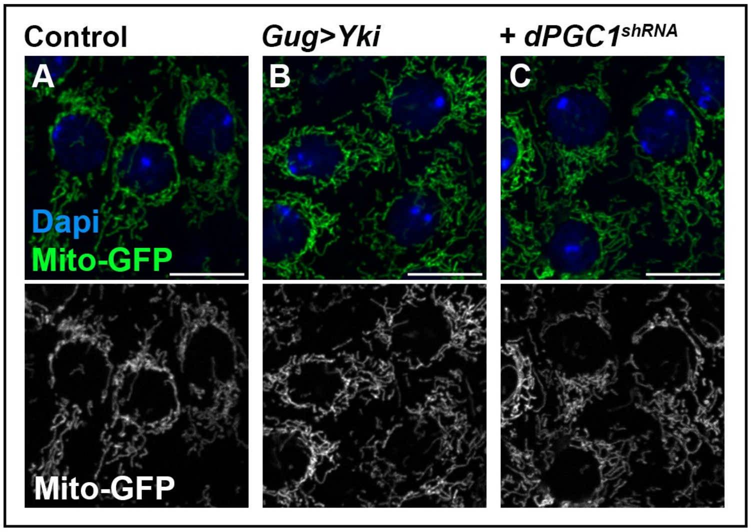

6. In Fig. 3I: what is really the mitochondrion? It would be good to outline the region(s) that was/were measured.

To improve clarity, we have repeated the electron microscopy (EM) analysis and now provide representative images that more clearly illustrate mitochondrial morphology in the different genotypes analyzed. These updated images presented in Fig 4 better highlight the structural alterations observed upon genetic manipulation and help clarify the basis for our morphological assessments.

We have extended our analysis and have assessed mitochondrial ultrastructure in Yki + dPGC1-RNAi wing disc tumors, with and without dMfn1 downregulation—the most upregulated mitochondrial fusion gene in this tumor context. In Yki + dPGC1-RNAi tumors, mitochondria appeared more elongated, consistent with increased fusion. Upon dMfn1 depletion, we observed a dramatic shift in mitochondrial morphology: mitochondria became larger and more rounded, with disrupted cristae and onion-like structures, indicative of compromised mitochondrial integrity and function (see new Fig 4).

7. A quantification of RNAi and overexpression efficiencies of the different transgenes in Fig. 3 is required.

To assess the efficiency of RNAi-mediated knockdown and transgene overexpression, we performed quantitative PCR (qPCR) using the ubiquitous Actin-Gal4 driver. While we acknowledge that this driver does not replicate the spatial specificity of the periodic membrane Gal4 driver used in the experiments shown in Figure 3 (Gug-Gal4), the latter targets a very limited number of cells within the imaginal disc, making reliable qPCR quantification unfeasible.

Using Actin-Gal4 allows us to obtain a relative and informative measure of transgene efficiency across the different constructs. These data confirm effective knockdown and overexpression of the relevant genes and are now included in Figure S2.

8. In Fig. 4: what is the phenotype when miro is over-expressed in combination with Yki? Or when it is knocked down in the ap>Yki-dPGC1 background? This was the gene tested in Fig. 3 with a clear mitochondrial phenotype

To address whether miro contributes to Yki-mediated tumor growth, we performed the requested experiments and now include the results in the revised manuscript (see updated Results section, lines 374-377, and new Fig. S11).

Our data show that overexpression of miro in combination with Yki does not lead to a significant increase in tissue growth or tumor-like phenotypes, in contrast to the effects observed with dMfn or Opa1 overexpression. Similarly, knockdown of miro in the ap>Yki-dPGC1-RNAi background did not suppress tumor growth, indicating that miro is not required for the enhanced proliferation observed in this context.

These findings suggest that, although miro influences mitochondrial morphology in normal wing discs (as shown in Fig. 3), its role in tumorigenesis is distinct from that of dMfn and Opa1. We have revised the manuscript to clarify the gene-specific contributions of mitochondrial fusion

regulators to Yki-driven tumorigenesis. This distinction underscores the complexity of mitochondrial dynamics and highlights that not all fusion-related genes exert the same functional impact in oncogenic settings.

How does the mitochondrial morphology in the wing disc peripodial epithelium look like in Gug>Opa1RNAi or Gug>Opa1 discs?

To assess the impact of Opa1 on mitochondrial morphology in the peripodial epithelium of the wing disc, we used the Gug-GAL4 driver to either overexpress or knock down Opa1. Our analysis revealed that Opa1 overexpression led to slightly elongated mitochondria, but did not result in extensive network formation, suggesting a modest enhancement of inner membrane fusion. In contrast, Opa1 knockdown caused clear mitochondrial fragmentation, closely resembling the phenotype observed upon dMfn depletion. These results shown in Fig 3 are consistent with the distinct roles of Opa1 and dMfn in regulating mitochondrial fusion: Opa1 primarily modulates inner membrane fusion and cristae architecture, while dMfn drives outer membrane fusion and network connectivity.

The corresponding data are presented in Figure 3F, G, and quantified in Figure S9, alongside experiments manipulating other genes involved in mitochondrial dynamics.

Why have the authors switched between the ap>Yki+dPGCRNAi and the ap>Yki+dPGC1shRNA lines? It would be important to have this series of experiments in the same backgrounds, as KD efficiencies are different (Fig. S1C).

The primary reason for switching between the dPGC1-RNAi and dPGC1-shRNA lines was practical: the chromosomal insertion sites of the transgenes made certain genetic combinations more feasible with one line over the other. This flexibility significantly facilitated our experimental design and analysis.

To address concerns regarding knockdown efficiency, we performed a comparative analysis using the ubiquitous actin-GAL4 driver, rather than MS1096-GAL4, which exhibits patchy and dynamic expression in the wing imaginal disc. This allowed us to obtain a more consistent and interpretable measure of mRNA downregulation for both transgenes. Our results show that both lines achieve comparable levels of knockdown, as shown in Figure S2.

9. Fig. 5A: proper quantification of Western Blot signals is required. I do not agree that Cyclin E protein levels are elevated in ap>Yki or ap>Yki+dPGC1 discs. Even at the mRNA levels the increase in expression is rather weak. From these results nothing can be concluded.

We have repeated the Western blot analysis using seven independent membranes to ensure robust quantification of Cyclin E levels in ap>Yki and ap>Yki+dPGC1-RNAi wing discs (Fig 6).

Although the increase in Cyclin E protein levels is subtle, it is consistent across replicates and statistically significant. We have now included the quantification of these Western blot signals in

the revised Figure 6, which supports the conclusion that Cyclin E levels are elevated in ap>Yki+dPGC1 discs.

We hope this additional data addresses the reviewer's concern and strengthens the interpretation of our results.

10. Knock-down efficiencies for dap and CycE needs to be quantifiec (Fig. 5H-N). Although the rescue experiment with CycE knock down is from the phenotype convincing, it is nonetheless puzzling, as CycE is accodring to Fig. 5A+B hardly upregulated. An independent CycE RNAi line would be useful.

We have quantified the knockdown efficiency of the dap-RNAi line, and the results are included in Figure S13.

Regarding Cyclin E, we would like to clarify that we did not use an RNAi line in this experiment. Instead, we employed the CycE-05306 mutant allele in a heterozygous background, which is expected to reduce Cyclin E levels by approximately 50%. The CycE-05306 allele in Drosophila melanogaster is a loss-of-function allele of the Cyclin E gene. This allele carries a P-element insertion in the first intron of the CycE gene, which disrupts normal transcription and reduces Cyclin E expression. In a heterozygous background, as used in your experiments, CycE-05306/+ is expected to reduce Cyclin E levels by approximately 50%, which is typically sufficient to observe genetic interactions or sensitized phenotypes without affecting normal development. This makes it a valuable tool for studying gene dosage effects, particularly in tumor models where Cyclin E activity may be rate-limiting.

Importantly, this partial reduction does not impair normal tissue growth, but it strongly limits tumor growth in the context of Yki overexpression combined with dPGC1 downregulation, as shown in Figure 6. This selective sensitivity highlights the functional importance of Cyclin E in supporting oncogenic growth driven by Yki and dPGC1 depletion. We believe this provides compelling evidence for Cyclin E's role in this tumor model.

Reviewer #3 (Significance (Required)):

Strengths and Limitations of the Study
Strengths
Innovative Focus on Mitochondrial Dynamics and Oncogenesis:
The study provides compelling evidence linking mitochondrial dynamics, particularly hyperfusion, to tumorigenesis in Drosophila. The identification of dPGC1 as a context-dependent tumor suppressor adds novel insights into the interplay between metabolism and oncogenesis.
Comprehensive Use of Drosophila as a Model System:
The study leverages the genetic tractability of Drosophila, allowing precise manipulation of mitochondrial regulators and signaling pathways. The use of wing imaginal discs as a model for tumor growth is well-established and appropriate.

Integration of Morphological and Genetic Data:
The manuscript combines confocal imaging, electron microscopy, and genetic tools to demonstrate the role of dPGC1 in regulating mitochondrial dynamics, Cyclin E levels, and tissue overgrowth.
Relevance to Cancer Biology:
The findings address key hallmarks of cancer, including deregulated metabolism, genomic instability, and cell cycle misregulation. The study's exploration of these processes in a simple model organism provides a strong basis for translating findings to mammalian systems.

Limitations
Validation of RNAi and Overexpression Efficiency:
The knockdown efficiency of dPGC1 on the mRNA level is only moderate (30-50%), and protein-level validation is missing. Without this, the study cannot conclusively demonstrate the role of dPGC1 in normal development or tumorigenesis.
Incomplete Mechanistic Insights:
The manuscript identifies Cyclin E as a potential driver of tumor growth but does not adequately explore how mitochondrial hyperfusion leads to Cyclin E regulation (e.g., post-transcriptional mechanisms or protein stability).
Inconsistencies in Experimental Backgrounds:
The study uses different RNAi/shRNA lines and driver combinations inconsistently across experiments, making it difficult to compare results directly. This variability undermines the robustness of the conclusions.
Limited Functional Analysis of Mitochondria:
While mitochondrial morphology is well-characterized, functional assays (e.g., membrane potential or ATP production) are missing. These would confirm the impact of hyperfusion on cellular energetics and oncogenesis.

In the revised manuscript, we have addressed each of the concerns raised.

In addition to that, in the revised version of the manuscript, we have included new experiments to assess mitochondrial functionality in tumors co-expressing Yki and dPGC1-RNAi. Specifically, we analyzed the Mitochondrial Membrane Potential (MMP). We used TMRE staining to evaluate MMP, a key indicator of mitochondrial integrity and oxidative phosphorylation capacity. Our analysis revealed no significant differences in MMP between Yki tumors and Yki + dPGC1-RNAi tumors, suggesting that mitochondrial membrane potential is preserved despite the observed morphological abnormalities. These results are shown in Fig S6. In the text it is discussed in lines 233-243.

Contribution to Existing Literature
The study makes a significant contribution to the growing body of literature on the metabolic regulation of cancer by identifying dPGC1 as a tumor suppressor modulating mitochondrial dynamics. Previous work has established the dual roles of mammalian PGC1α in promoting or

suppressing cancer depending on context. This study adds depth by demonstrating similar context-dependent effects in a simpler model organism, facilitating further exploration of the molecular mechanisms involved.

By linking mitochondrial fusion, Yki signaling, and Cyclin E regulation, the manuscript aligns with and expands upon research on Hippo pathway regulation, cancer metabolism, and mitochondrial biology. The findings highlight the importance of integrating metabolic and signaling networks in understanding oncogenesis.

Community Selection
The current form of the manuscript is best suited for a specialized audience, particularly mitochondrial biologists, Drosophila researchers, and Hippo pathway specialists. To engage a broader community, additional work linking these findings to mammalian models or human cancer biology would be necessary.
