# Supplementary material for: Control of mitochondrial dynamics by the metabolic regulator dPGC1 limits Yorkie-induced oncogenic growth in Drosophila
Source: PLoS Biol. 2025 Dec 4;23(12):e3003523. doi: 10.1371/journal.pbio.3003523 (PMC12697956; doi:10.1371/journal.pbio.3003523)
Supplement: Attachment — Submitted filename: Response_to_Reviewers.pdf [file pbio.3003523.s020.pdf]

RESPONSE TO REVIEWER 3

Rev. 3:
This revised manuscript represents a substantial step forward and now delivers a coherent and well-substantiated story. The authors convincingly demonstrate that the conserved co-activator dPGC1 functions as a context-dependent tumor suppressor in Drosophila, constraining Yki-driven oncogenic growth through control of mitochondrial dynamics and Cyclin E regulation. The combination of genetic precision, structural analysis (confocal and EM), and functional assays y+ ields a mechanistically grounded picture linking mitochondrial remodeling to cell-cycle control. The topic is timely and relevant across developmental biology, cancer metabolism, and Hippo signaling.

Major strengths
1. Rigorous revision and new experiments. The inclusion of loss-of-function alleles ($dPGC1^{1}$, $Wts^{-}/^{-}$), improved RNAi validation using act-GAL4, and expanded EM/confocal analyses greatly enhance robustness.

2. Mechanistic insight. The demonstration that Cyclin E dosage genetically modifies tumor growth (via CycE05306/+) establishes a functional link between mitochondrial dynamics and proliferation.

3. Context specificity. The observation that dPGC1 depletion does not potentiate EGFR or InR overgrowthconvincingly shows that this interaction is Yki-specific, addressing a key conceptual gap.

4. Integration with prior literature. The authors now situate their work alongside Nagaraj et al. 2012 and Spiegelman-lab studies, acknowledging precedent while delineating novelty.

5. Improved clarity and presentation. Figures now include quantified PH3, Mmp1, F-actin, and Western replicates; the narrative flow is logical and accessible.

Remaining issues for a final, publication-ready version
All points below are minor yet important refinements that will raise the manuscript to PLOS Biology's highest standard:

1. In the absence of an antibody, please state explicitly in Methods and Discussion that conclusions rest on transcript and genetic evidence, and discuss the limitation.

We have addressed this point by explicitly stating in the Discussion section that our conclusions regarding dPGC1 are based on transcript and genetic evidence due to the lack of a specific antibody. We also acknowledge this as a limitation of the study. The following sentence has been added to the end of the first paragraph in the Discussion: "Due to the lack of a specific antibody against dPGC1, our conclusions are based on transcript and genetic evidence, which limits direct assessment of protein-level regulation in this context."

Additionally, we have included a note in the Materials and Methods section to clarify this point: “Note: Due to the absence of a specific antibody against dPGC1, all conclusions regarding its role are based on transcript and genetic evidence.”

These changes are now reflected in the revised manuscript.

2. Supplement EM length data with an additional shape descriptor (aspect ratio or circularity) to distinguish elongation from swelling; even approximate metrics will strengthen claims.

We agree that including shape descriptors is important for distinguishing mitochondrial elongation from swelling. As part of our previous revision, we already incorporated quantification of mitochondrial aspect ratio in Figure 4E, alongside perimeter measurements in Figure 4D. This analysis provides a complementary metric to support our interpretation of mitochondrial remodeling in the tumor context.

3. The TMRE analysis is valuable; ensure inclusion of numerical quantification, n-values, and statistics in the figure legend, and briefly discuss how preserved MMP may indicate compensatory metabolic adjustments.

We acknowledge the importance of including detailed quantification and statistical information. As part of the previously revised version of the manuscript, the figure legend for Figure S6 already includes TMRE intensity quantification, n-values, and statistical analysis (Mann–Whitney test). We have double-checked to ensure this information is clearly presented.

Related to the second comment, we have addressed this point by expanding the relevant section in the Results. Specifically, we now include the following sentence at the end of the paragraph titled “Mitochondrial Membrane Potential in Yki-Driven Tumors”:

“This preservation of MMP may reflect compensatory metabolic adjustments that help maintain mitochondrial function despite structural remodeling, allowing tumor cells to sustain their energetic demands.”

This addition acknowledges the potential metabolic implications of preserved mitochondrial membrane potential in the tumor context.

4. Expand the Discussion by speculating, succinctly, on plausible post-transcriptional mechanisms (e.g. altered degradation, ROS-mediated signaling) linking mitochondrial state to Cyclin E stabilization.

In response, we have expanded the Discussion to include plausible post-transcriptional mechanisms linking mitochondrial state to Cyclin E stabilization. Specifically, we now note that Cyclin E mRNA levels remain unchanged, and propose that mitochondrial dysfunction may activate retrograde signaling via ROS, which modulates cell cycle regulators such as p53 and Dacapo, thereby influencing Cyclin E

turnover (see revised Discussion). These additions provide a mechanistic framework consistent with our observations in Yki + dPGC1 tumors.

5. Provide a table detailing RNAi/shRNA lines, insertion sites, drivers, and efficienciesto aid reproducibility and transparency.

A Table showing this information is present in the revised version of our manuscript as S1 Data.

6. A careful proof for minor grammatical inconsistencies (articles, tense) will further polish the manuscript.

We have carefully proofread the manuscript to address minor grammatical inconsistencies, including article usage and verb tense. All necessary corrections have been implemented to further polish the text.

Overall assessment
The authors have been highly responsive and thorough, transforming an interesting initial study into a rigorous and conceptually compelling paper. The work establishes mitochondrial dynamics as an integral layer of Hippo pathway regulation and identifies dPGC1 as a safeguard against Yki-induced tumorigenesis - a finding with clear translational resonance.
Recommendation: Accept pending minor revision.

With these final adjustments, the manuscript will stand as a benchmark study for integrating metabolic control, organelle dynamics, and growth signaling in vivo.
